# Engineering the β-Carotene Metabolic Pathway of Microalgae *Dunaliella* To Confirm Its Carotenoid Synthesis Pattern in Comparison To Bacteria and Plants

**DOI:** 10.1128/spectrum.04361-22

**Published:** 2023-01-31

**Authors:** Hao-Hong Chen, Ming-Hua Liang, Zhi-Wei Ye, Yue-Hui Zhu, Jian-Guo Jiang

**Affiliations:** a School of Food Science and Engineering, South China University of Technology, Guangzhou, China; b Department of Bioengineering, Imperial College London, London, United Kingdom; c College of Food Science, South China Agricultural University, Guangzhou, China; d Washington University School of Medicine, St. Louis, Missouri, USA; University of Pennsylvania

**Keywords:** *Dunaliella salina*, carotenoid metabolic pathway, lycopene, β-carotene, gene expression, plasmid construction

## Abstract

*Dunaliella salina* is the most salt-tolerant eukaryote and has the highest β-carotene content, but its carotenoid synthesis pathway is still unclear, especially the synthesis of lycopene, the upstream product of β-carotene. In this study, *DsGGPS*, *DsPSY*, *DsPDS*, *DsZISO*, *DsZDS*, *DsCRTISO*, and *DsLYCB* genes were cloned from *D. salina* and expressed in Escherichia coli. A series of carotenoid engineering E. coli strains from phytoene to β-carotene were obtained. *ZISO* was first identified from *Chlorophyta*, while *CRTISO* was first isolated from algae. It was found that DsZISO and DsCRTISO were essential for isomerization of carotenoids in photosynthetic organisms and could not be replaced by photoisomerization, unlike some plants. DsZDS was found to have weak beta cyclization abilities, and DsLYCB was able to catalyze 7,7′,9,9′-tetra-*cis*-lycopene to generate 7,7′,9,9′-tetra-*cis*-β-carotene, which had not been reported before. A new carotenoid 7,7′,9,9′-tetra-*cis*-β-carotene, the beta cyclization product of prolycopene, was discovered. Compared with the bacterial-derived carotenoid synthesis pathway, there is higher specificity and greater efficiency of the carotenoid synthesis pathway in algae. This research experimentally confirmed that the conversion of phytoene to lycopene in *D. salina* was similar to that of plants and different from bacteria and provided a new possibility for the metabolic engineering of β-carotene.

**IMPORTANCE** The synthesis mode of all *trans*-lycopene in bacteria and plants is clear, but there are still doubts in microalgae. *Dunaliella* is the organism with the highest β-carotene content, and plant-type and bacterial-type enzyme genes have been found in its carotenoid metabolism pathway. In this study, the entire plant-type enzyme gene was completely cloned into Escherichia coli, and high-efficiency expression was obtained, which proved that carotenoid synthesis of algae is similar to that of plants. In bacteria, CRT can directly catalyze 4-step continuous dehydrogenation to produce all *trans*-lycopene. In plants, four enzymes (PDS, ZISO, ZDS, and CRTISO) are involved in this process. Although a carotenoid synthetase similar to that of bacteria has been found in algae, it does not play a major role. This research reveals the evolutionary relationship of carotenoid metabolism in bacteria, algae, and plants and is of methodologically innovative significance for molecular evolution research.

## INTRODUCTION

*Dunaliella salina*, a marine unicellular green alga, is the most salt-tolerant eukaryote with the highest natural β-carotene content known at present (up to 10% of the dry weight) and is a model organism both for osmoregulation and carotenoid metabolism research (1–3). However, the identification and functional verification of its β-carotene pathway synthases are still incomplete.

**FIG 1:**
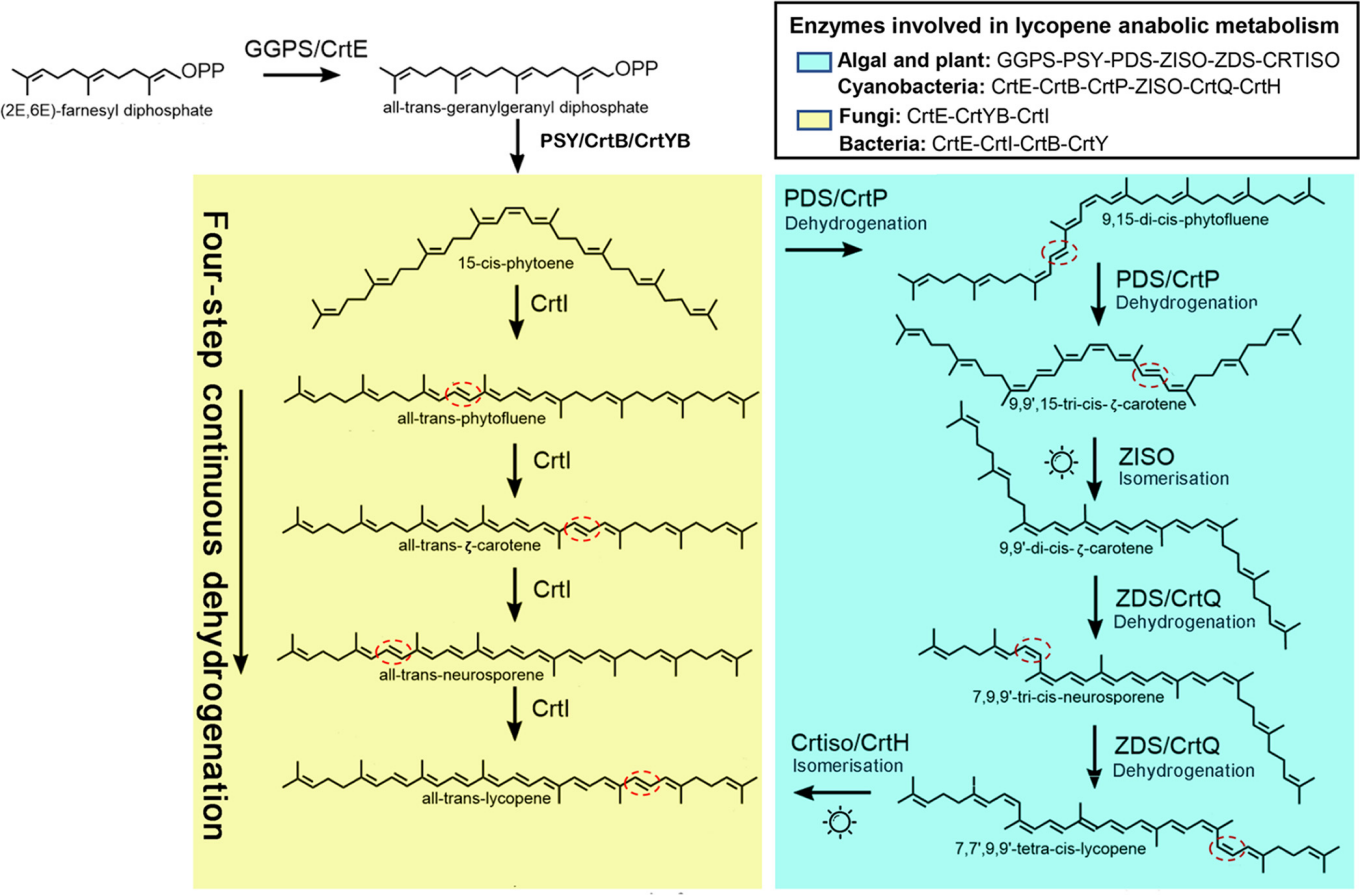
Biosynthesis of carotenoids in bacteria, fungi, cyanobacteria, algae, and plants. Bacteria can synthesize C40 carotenoids via different biosynthetic pathways. Bacterial CrtI can catalyze the three-step or four-step desaturations, while fungal CrtI can catalyze the four-step or five-step desaturations.

The biosynthesis of carotenoids is an important branch of the isoprenoid metabolic pathway. Phytoene is the first carotenoid in this pathway, and lycopene is the center and branch point of this system (Fig. 1). Lycopene gradually forms more than 700 known carotenoids after a series of branching metabolism by alpha- and beta-cyclization (4–6).

In bacteria and fungi, the conversion from phytoene to lycopene is catalyzed by a CrtI-type phytoene dehydrogenase to complete a four-step continuous dehydrogenation (7, 8). In plants, four key enzymes are involved in this process: phytoene desaturase (PDS) catalyzes the dehydrogenation of 15-*cis*-phytoene twice to successively produce 9,15-*cis*-phytoene and 9,15,9′-tri-*cis*-ζ-carotene. Subsequently, 9,15,9′-tri-*cis*-ζ-carotene is converted to 9,9′-di-*cis*-ζ-carotene by 15-*cis*-ζ-carotene isomerase (ZISO) or photoisomerization. ζ-Carotene desaturase (ZDS) then dehydrogenates 9,9′-di-*cis*-ζ-carotene to 7,9,9′-tri-*cis*-neurosporene and 7,7’,9,9’-tetra-*cis*-lycopene successively. Finally, carotenoid isomerase (CRTISO) catalyzes the production of all-*trans*-lycopene from 7,7′,9,9′-tetra-*cis*-lycopene (prolycopene) (4).

It is generally believed that the carotenoid pathway of green algae is consistent with that of higher plants, but there are still many problems to be solved (9–11). In plant genetic engineering, a bacterial *CrtI* gene can be used to replace the four genes of *PDS*, *ZISO*, *ZDS*, and *CRTISO* of plants to achieve carotenoid accumulation. The bacterial CrtI gene is introduced in carotenoid-rich “golden rice” to achieve the conversion of phytoene to lycopene (12, 13). It has not been confirmed whether green algae have the four genes that can be replaced by the bacterial *CrtI* gene or whether these four enzymes have the same function as plants.

The aim of this study was to clone all the key enzyme genes of the β-carotene metabolic pathway in *D. salina*, including geranylgeranyl pyrophosphate synthase (*GGPS*), phytoene synthase (*PSY*), *PDS*, *ZISO*, *ZDS*, *CRTISO*, and lycopene β-cyclase (*LYCB*), and construct these genes into Escherichia coli to express them completely. By observing the expression efficiency of β-carotene and various intermediate carotenoids upstream, the application potential of β-carotene engineering E. coli strains constructed by *D. salina*’s carotenoid synthesis gene was judged. At the same time, according to the intermediate carotenoid products, consistencies or differences between lycopene synthesis in green algae and plants were confirmed. The functions of ZISO and CRTISO in green algae were further elucidated by comparing light and dark conditions.

## RESULTS AND DISCUSSION

### Isolation of the carotenoid genes from *D. salina*.

**FIG 2:**
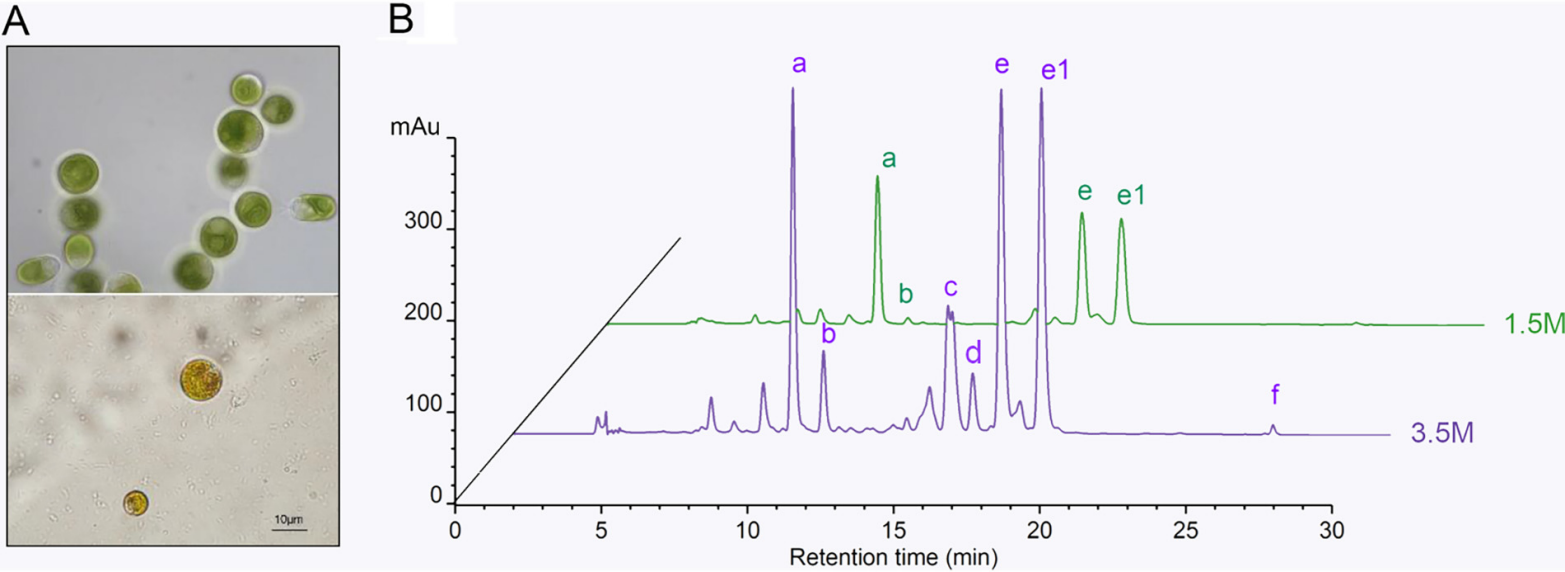
(A) Images of cells at 1.5 M NaCl (top) and 3.5 M NaCl (bottom). (B) Carotenoid analysis of *D. salina* CCAP 19/18 at 1.5 M and 3.5 M NaCl. The following are the peak assignments: a, lutein; b, zeaxanthin; c, α-cryptoxanthin; d, α-carotene; e, all-*trans*-β-carotene; e1, 9-*cis*-β-carotene; f, γ-carotene. mAu, arbitrary unit.

*D. salina* can grow in saturated saline. Its optimum salinity is 1.5 to 2.0 M NaCl. When the salinity exceeds 3.5 M NaCl, a large amount of β-carotene will accumulate, making the cells yellowish red (Fig. 2).

To identify the *DsGGPS*, *DsPSY*, *DsPDS*, *DsZISO*, *DsZDS*, *DsCRTISO*, and *DsLYCB* genes in *D. salina*, homology searches of the genomic sequences of *D. salina* were performed on the Phytozome platform using the corresponding amino acid sequences in Arabidopsis thaliana (14, 15). Candidate *crts* (carotenogenic enzymes) genes were found and isolated from the cDNAs of *D. salina*, and the results are shown in Table S1 available at https://www.biosynnatlab.com/wp-content/uploads/2022/12/Supplemental-Material.pdf. The function of ZISO in *Euglena* has been identified, but not in *Chlorophyta*, while CRTISO was first isolated and identified from algae. The initial ATG of each gene was verified by 5′ rapid amplification of cDNA ends (RACE) PCR, and the termination codon of each gene was verified by 3′ RACE PCR.

The length of the coding DNA sequence (CDS) of the *crts* gene, the length of the coding amino acids, and the similarity with *Arabidopsis*-related genes are listed in Table S1 at https://www.biosynnatlab.com/wp-content/uploads/2022/12/Supplemental-Material.pdf. Among them, *DsGGPS*, *DsPSY*, *DsZISO*, *DsZDS*, *DsCRTISO-homo1*, *DsCRTISO-homo2*, and *DsLYCB* were consistent with the sequence information provided by the Phytozome platform, while *DsPDS* and *DsCRTISO* were incomplete in Phytozome. Compared with the functional Crts enzymes of *A. thaliana*, the deduced amino acid sequences of *D. salina* were highly conserved (50 to 70%), except for the N-terminal region, which implied potential functional similarities among these proteins.

Two transmembrane domains of each enzyme were predicted using the TMHMM program (http://www.cbs.dtu.dk/services/TMHMM/), which were present in typical plastid-targeting proteins (http://www.cbs.dtu.dk/services/ChloroP/), indicating the plastid localization of *D. salina* Crts protein.

### Construction and expression of the *D. salina* β-carotene synthesis pathway in E. coli.

**FIG 3:**
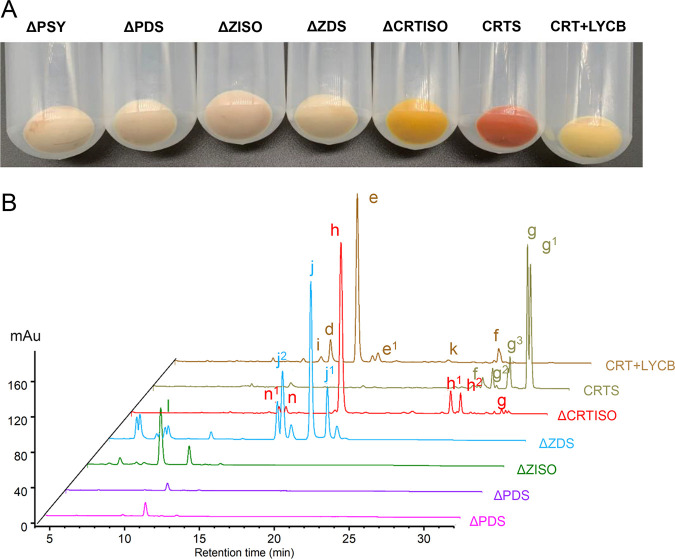
Carotenoid composition of various engineering Escherichia coli. (A) Colors of E. coli (engineered) strains harboring different combinations of plasmids. (B) HPLC analysis of the carotenoids produced in E. coli cells harboring different combinations of plasmids. The following are the peak assignments: d, α-carotene; e, all-*trans*-β-carotene; e1, 9-*cis*-β-carotene; f, γ-carotene; g, all-*trans*-lycopene; g1, lycopene isomer1; g2, lycopene isomer2; g3, lycopene isomer3; h, 7,7′,9,9′-tetra-*cis*-lycopene (prolycopene); h1, prolycopene isomer1; h2, prolycopene isomer2; i, ε-carotene; j, 9,9′-di-*cis*-ζ-carotene; j1, ζ-carotene isomer1; j2, ζ-carotene isomer2; k, δ-carotene; l, 9,9′,15-tri-*cis*-ζ-carotene; n, 7,9,9′-tri-*cis*-neurosporene; n1, neurosporene isomer1.

The function of *D. salina* Crts was confirmed by using a heterologous E. coli expression system. Fig. 3A shows a series of strains that were cultured under dark conditions at 28°C and centrifuged after isopropyl-β-d-thiogalactopyranoside (IPTG) induction for 48 h. It can be seen that the absence of *DsPSY*, *DsPDS*, and *DsZISO* all formed pale yellow bacteria, The Δ*ZDS* strain produced beige bacteria, the Δ*CRTISO* strain formed golden yellow bacteria, the CRTS strain formed red bacteria, and the CRT+LYCB strain formed yellowish bacteria, which indicated that these enzymes could be normally expressed in E. coli and had catalytic activity. It could also be seen from Fig. 3B that a lack of any one of the *crt* genes could not produce β-carotene.

The carotenoid compositions of Δ*PSY*, Δ*PDS*, Δ*ZISO*, Δ*ZDS*, Δ*CRTISO*, CRTS, and CRT+LYCB strains were detected by high-performance liquid chromatography (HPLC) analysis. The retention time and characteristic absorption spectrum of each carotene are shown in Fig. 3B and in Fig. S1 and Table S2 at https://www.biosynnatlab.com/wp-content/uploads/2022/12/Supplemental-Material.pdf. The detected 9,15,9′-tri-*cis*-ζ-carotene and 9,9′-di-*cis*-ζ-carotene are consistent with the characteristic peaks in the HPLC results of the E. coli strain containing pAC-ZETAipi (Fig. S2 at https://www.biosynnatlab.com/wp-content/uploads/2022/12/Supplemental-Material.pdf). The liquid chromatogram peak with 9,15,9′-tri-*cis*-ζ-carotene (l) as the main substance was detected from Δ*ZISO* at a 400-nm band. Likewise, a liquid chromatogram peak with 9,9′-di-*cis*-ζ-carotene (j) as the main substance was detected in Δ*ZDS* at a 400-nm band for 17.29 min, and a liquid chromatogram peak with 7,7′,9,9′-tetra-*cis*-lycopene (h) as the main substance was detected in Δ*CRTISO* at a 450-nm band for 18.06 min. CRTS and CRT+LYCB strains formed all-*trans*-lycopene and β-carotene at 29.18 and 16.27 min, respectively. Among them, there were more isomers of lycopene, which had a great relationship with 13 carbon-carbon double bonds in lycopene.

### Functional catalytic properties of *DsLYCB*.

Through further analysis of the CRT+LYCB strain HPLC results in Fig. 3B, it was found that in addition to the production of β-carotene, δ-carotene, ε-carotene, γ-carotene, and α-carotene were also generated. Many studies have reported the functional identification and regulatory mechanism of LCYB in *D. salina* (16, 17). But little research has reported that LYCB can catalyze all-*trans*-lycopene to produce α-carotene, δ-carotene, and ε-carotene (18).

In the lycopene cyclization process, it may be that C-2 dehydrogenates under the effect of the cofactor NAD, making C-1 exhibit electrophilicity, attacking C-6, and then C-4, C-5, and C-6 form delocalized π bonds, which are conjugated with the C-2 dumbbell electronic cloud to form larger delocalized π bonds (18, 19). A β-ring is formed when C-6 hydrogen is transferred to the C-2 position, and an ε-ring is formed when C-4 hydrogen is transferred to the C-2 position. Under the catalysis of DsLYCB, C-6 hydrogen tends to be transferred to the C-2 position, so most of them form β-carotene (84%) and a small amount of ε-ring (16%). This agreed with our previous report suggesting that DbLCYB is bifunctional in *Dunaliella bardawil* (20). Interestingly, maize LcyE (ZmLcyE) also showed a low level of β-monocyclase activity (21). But we found that there was another cyclase in *Dunaliella* (DbLCYE) that only showed ε-cyclase activity (20). It has been reported that a conserved motif (designated the extended motif B) of lycopene cyclases determines the formation of β- and ε-ionone groups (22).

**FIG 4:**
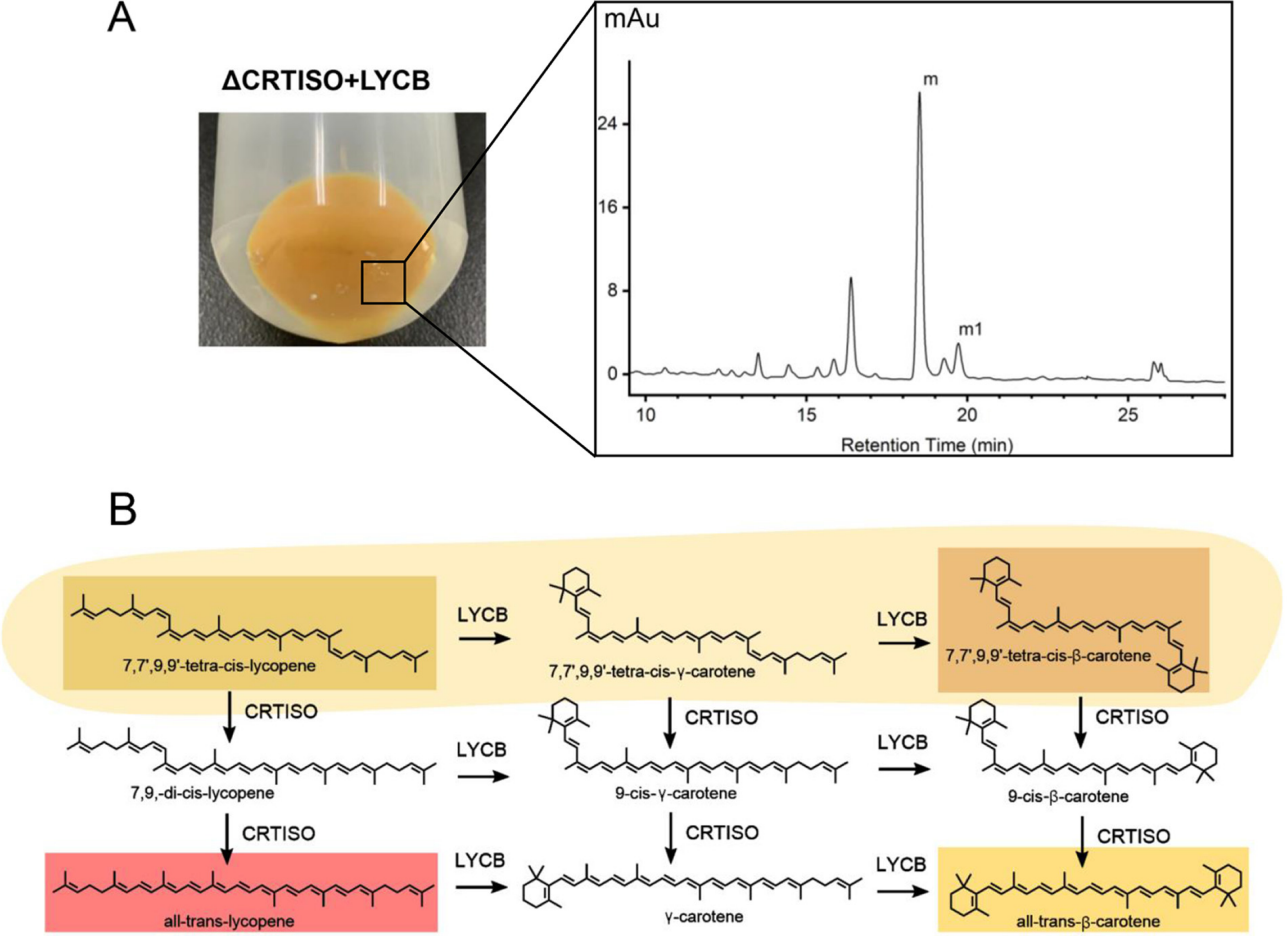
Functional characteristics of LYCB from *D. salina.* (A) HPLC scans of carotenoid extracts from Δ*CRTISO*-LYCB; peaks: m, 7,7′,9,9′-tetra-*cis*-lycopene; m1, 7,7′,9,9′-tetra-*cis*-lycopene isomer. (B) CRTISO and LYCB involved in the probable pathway of β-carotene biosynthesis.

Interestingly, analysis of the liquid chromatogram of the Δ*CRTISO*+*LYCB* strain in Fig. 4A revealed the production of a new carotenoid not previously reported, which has a distinctly different retention time and characteristic absorption spectrum from that of β-carotene (Fig. 2; Table S2 https://www.biosynnatlab.com/wp-content/uploads/2022/12/Supplemental-Material.pdf). It has been reported that neurosporene can be directly cyclized to β-zeacarotene under the catalysis of LYCB (18). Only one end of the ψ-end group with C-7 and C-8 dehydrogenation can be cyclized by LYCB, while the ψ-end group without dehydrogenation cannot be cyclized (18). By comparing the retention time and characteristic absorption spectrum, it was inferred that this new carotenoid was the β-cyclization product of prolycopene, namely, 7,7′,9,9′-tetra-*cis*-β-carotene. This new carotenoid implies a new way to synthesize β-carotene (Fig. 4B).

All-*trans*-β-carotene was not detected in the Δ*CRTISO*+*LYCB* strain, indicating that the *cis* structure at C-7 and C-9 was a bond-energy-stable structure, and no isomerization occurred automatically during the cyclization process (Fig. 4B). In addition, the *cis* structure of C-7 and C-9 has a larger spatial conformation, making it more difficult to enter the catalytic channel, and this location is unlikely to have an isomeric catalytic site (23). Therefore, the catalytic process of the *cis* structure of C-7 and C-9 is consistent with the cyclization process of all-*trans*-lycopene; dehydrogenation occurs at the 2 position, and then the 1 position attacks C-6 (Fig. S3 at https://www.biosynnatlab.com/wp-content/uploads/2022/12/Supplemental-Material.pdf).

The traditional view is that LYCB has only *trans*-ψ-end group catalytic ability (18). This study found that DsLYCB also has *cis* catalytic ability. This result also explained the reason that the contents of 9-*cis*-β-carotene and all-*trans*-β-carotene in *D. salina* are so close (Fig. 2B). The isomerization of DsCRTISO in the synthesis of β-carotene is not complete, leading to the accumulation of 9-*cis*-carotene. However, there are different internal environments between E. coli and chloroplasts of *Dunaliella*. More accurate verification requires the removal of *DsLYCB* from *D. salina* by knockout technology.

### Functional catalytic properties of DsZISO and DsCRTISO.

Many reports believed that 9,9′,15-tri-*cis*-ζ-carotene and 7,7′,9,9′-tetra-*cis*-lycopene can be isomerized into 9,9′-di-*cis*-ζ-carotene and all-*trans*-lycopene, provided that they are directly exposed to light (24, 25). A study of *Arthrospira platensis* showed that light conditions could produce isomeric effects and performed the function of ZISO, but the CrtP of *A. platensis* itself has the function of ZISO isomerase (26). It is hard to say whether ZISO or CrtP was working.

**FIG 5:**
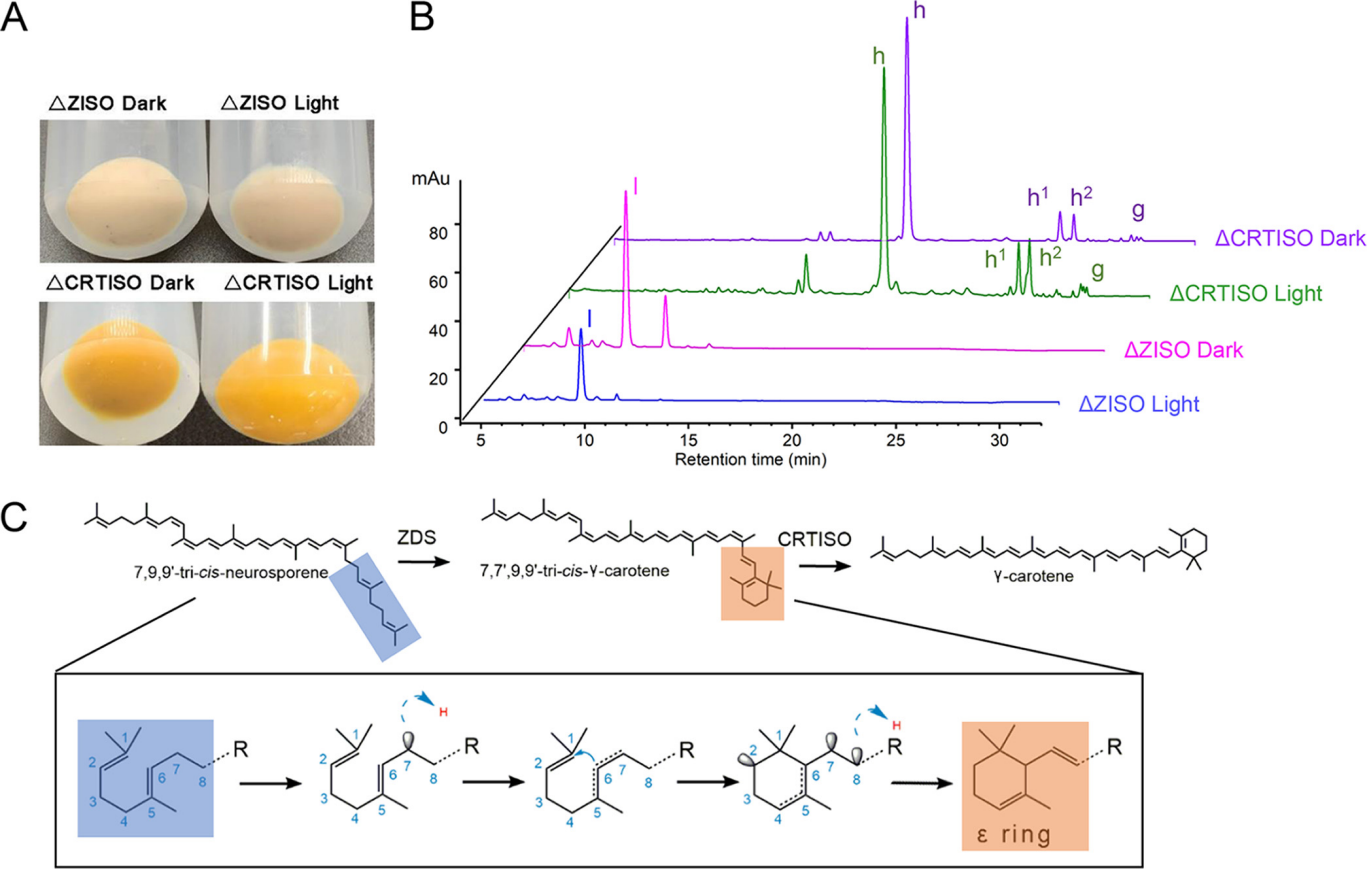
Functional identification of ZISO, CRTISO, and ZDS. (A) Colors of Δ*ZISO* and Δ*CRTISO* E. coli strains under light and dark culture conditions, respectively. (B) HPLC scans of carotenoid extracts showing no differences between light and dark culture of Δ*ZISO* and Δ*CRTISO* strains; peaks: g, all-*trans*-lycopene; h, 7,7′,9,9′-tetra-*cis*-lycopene (prolycopene); h1, prolycopene isomer1; h2, prolycopene isomer2; l, 9,9′,15-tri-*cis*-ζ-carotene. (C) Putative γ-carotene biosynthesis pathway catalyzed by ZDS and CRTISO.

Δ*ZISO* and Δ*CRTISO* strains were cultured under light and dark conditions at 28°C, respectively, and we found that light and dark conditions did not change the color of the bacteria (Fig. 5A). HPLC analysis also found that the peaks of Δ*ZISO* and Δ*CRTISO* strains cultured under dark and light conditions did not change (Fig. 5B). Under light conditions, 9,15,9′-tri-*cis*-ζ-carotene and 7,7′,9,9′-tetra-*cis*-lycopene in Δ*ZISO* and Δ*CRTISO* could not be well isomerized into 9,9′-di-*cis*-ζ-carotene and all-*trans*-lycopene to produce red lycopene mycelia. This result indicated that ZISO and CRTISO are necessary for the production of β-carotene by photosynthetic organisms, such as green algae and even plants. This result was consistent with that of Yu et al. (24) but differed from Elio et al. (27) who believed that light restores lycopene biosynthesis in *ZISO*-silenced fruits.

Due to the influence of the cell wall and stroma, light energy is greatly weakened in cells, resulting in 7,7′,9, 9′-tetra-*cis*-lycopene not being well isomerized. It should be noted that a small amount of all-*trans*-lycopene was detected in the Δ*CRTISO* strain cultured under light and dark conditions. It can be speculated that if CRTISO is removed, the green algae or plants may still survive. This may be the reason why some of the CRTISO-mutant plants can continue to survive despite being cultured in the dark (24, 27).

### Functional catalytic properties of DsZDS.

In plants, ZDS is a well-studied enzyme (28–31). ZDS catalyzed the production of prolycopene from 9,9′-di-*cis*-ζ-carotene, but liquid chromatogram analysis of the CRTS strain revealed the presence of a small amount of γ-carotene, which was not reported previously. It is speculated that this phenomenon may occur during DsZDS-mediated dehydrogenation at C-7 and C-9, and its catalytic process is shown in Fig. 5C. Dehydrogenation occurs first at C-7, followed by conjugation at C-5, C-6, and C-7. It is known that the 6-membered ring structure is more stable than other ring structures, so C-6 attacks C-1, and the hydrogen of the 5 position shifts to the 2 position to form the β-ring structure (32, 33). Because of the presence of DsCRTISO, the C-7 and C-9 positions were isomerized into *trans* structures, forming γ-carotene (Fig. 5C).

### Phylogenetic distribution of *crts*.

To study the phylogenetic distribution of *crts*, a genome-wide survey of phototrophic organisms of *crts* was investigated. It was found that *crts* homologs are widely distributed in the genomes of photosynthetic organisms, such as plants, algae, and cyanobacteria. Because DsGGPS, DsPSY, DsPDS, DsZDS, and DsLYCB are common carotenoid synthases, they will not be discussed here. Only DsZISO and DsCRTISO with relatively few reports were analyzed.

By analyzing the evolutionary tree of ZISO, it was found that DsZISO and ZISO of green algae form a family cluster. Interestingly, ZISO of cyanobacteria had closer homology with higher plants (Fig. S4A at https://www.biosynnatlab.com/wp-content/uploads/2022/12/Supplemental-Material.pdf), indicating that prokaryotes produced branches during their evolution into plants and green algae. DsCRTISO did not have high homology with the CRTISO of plants and CrtH of cyanobacteria and was far apart in classification (Fig. S4B at https://www.biosynnatlab.com/wp-content/uploads/2022/12/Supplemental-Material.pdf). The two homologous isomerases of DsCRTISO were closer to those of plants and cyanobacteria; in particular, DsCRTISO-homo1 was close to that of the cyanobacteria Nostoc piscinale. However, our functional validation found that DsCRTISO-homo1 and DsCRTISO-homo2 did not have isomerase activity; they could not isomerize prolycopene to lycopene. This is an important reason why CRTISO isomerase had not been validated in green algae.

Phytoene desaturases of different biological origins show functional diversity. In most cyanobacteria, algae, and higher plants, carotenoid biosynthetic pathways consist of multiple enzymes involved in lycopene formation, whereas in most microorganisms, only one enzyme, CrtI-type, is involved in the dehydrogenation of phytoene, such as in Blakeslea trispora, Xanthophyllomyces dendrorhous, and Rhodosporidium diobovatum, while Neurospora crassa CrtI catalyzes the five-step dehydrogenation of phytoene to all-*trans*-3,4-didehydrolycopene.

**FIG 6:**
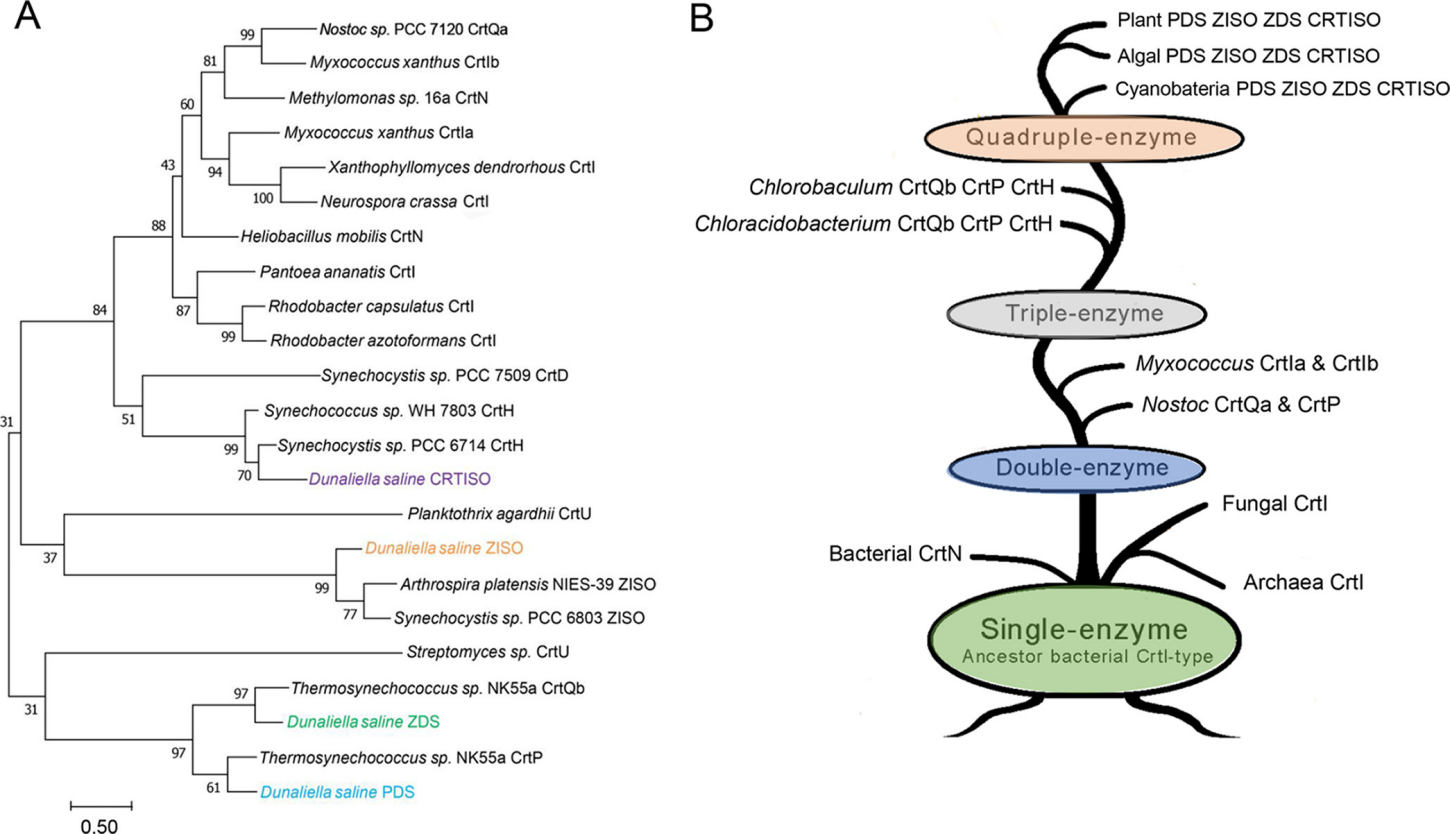
Evolutionary analysis of phytoene desaturase. (A) Phylogenetic tree analysis of phytoene dehydrogenases based on the amino acid sequences. The phylogenetic tree was constructed using the maximum-likelihood method of MEGA7 software with the JTT substitution model. Numbers associated with the branches were the bootstrap values (*n* = 1,000). Accession numbers of phytoene dehydrogenases protein sequences are shown in Table S5 at https://www.biosynnatlab.com/wp-content/uploads/2022/12/Supplemental-Material.pdf. (B) Evolutionary pathway analysis of phytoene dehydrogenases.

A phylogenetic tree was constructed based on protein sequence homology searches of phytoene desaturase from different sources, and the evolutionary pathway of the enzyme was analyzed (Table 1; Fig. 6A and B). The CrtI-type monoenzyme of a bacterial source may be the “common ancestor” of phytoene desaturase (Fig. 6B), which evolved into the *Haloarchaea* CrtI-type phytoene desaturase and the fungal CrtI-type phytoene desaturase (34). In addition, during the evolution of bacterial phytoene desaturase, substrate-specific alterations were exhibited, such as Staphylococcus aureus CrtN catalyzing C30 diapophytoene to generate C30 diapolycopene (35). The high homology of *Nostoc* CrtP and CrtQa with bacterial-derived CrtI-type phytoene desaturase suggests that *Nostoc* CrtP and CrtQa evolved from bacterial-derived CrtI-type to form the CrtP/CrtQa double enzyme system. *Nostoc* CrtQa evolved into the cyanobacterial *cis*-*trans* isomerases CrtH and CrtQb (36), resulting in the cyanobacterial CrtP/CrtQb/CrtH-type tri-enzyme system. Finally, The CrtP/CrtQb/CrtH-type tri-enzyme system has evolved into the more complex plant-derived PDS/ZDS/ZISO/CRTISO-type quadruple-enzyme system. It is noteworthy that the discovery of ZISO in cyanobacteria has recently been reported and has been shown to be widespread (26), but its presence is nonessential. In contrast, it has not been found in *Chlorobiaceae*, which is more evidence that the plant four-enzyme system evolved from cyanobacteria.

**TABLE 1:** Functional diversity of CrtI-type phytoene dehydrogenase

Type	Organism	Phytoene	ζ-Carotene	Neurosporene	Lycopene	Reference
One enzyme	*Rhodosporidium diobovatum*	CrtI (FAD dependent)				46
	Rhodobacter azotoformans					47
	Erwinia uredovora					38
	Neurospora crassa	Al-1 (NAD dependent)				48
Double enzyme	Myxococcus xanthus	CrtIa		CrtIb		49
Triple enzyme	Chlorobaculum tepidum	CrtP		CrtQb	CrtH	50
	*Chloracidobacterium*					26
Quadruple enzyme	*Cyanobacteria*	CrtP		CrtQa		36
			ZISO	CrtQb	CrtH	26
	Algal	PDS	ZISO	ZDS	CRTISO	In this study
	Plant					27

### Comparison between algal and bacterial carotenoid synthesis pathways.

**FIG 7:**
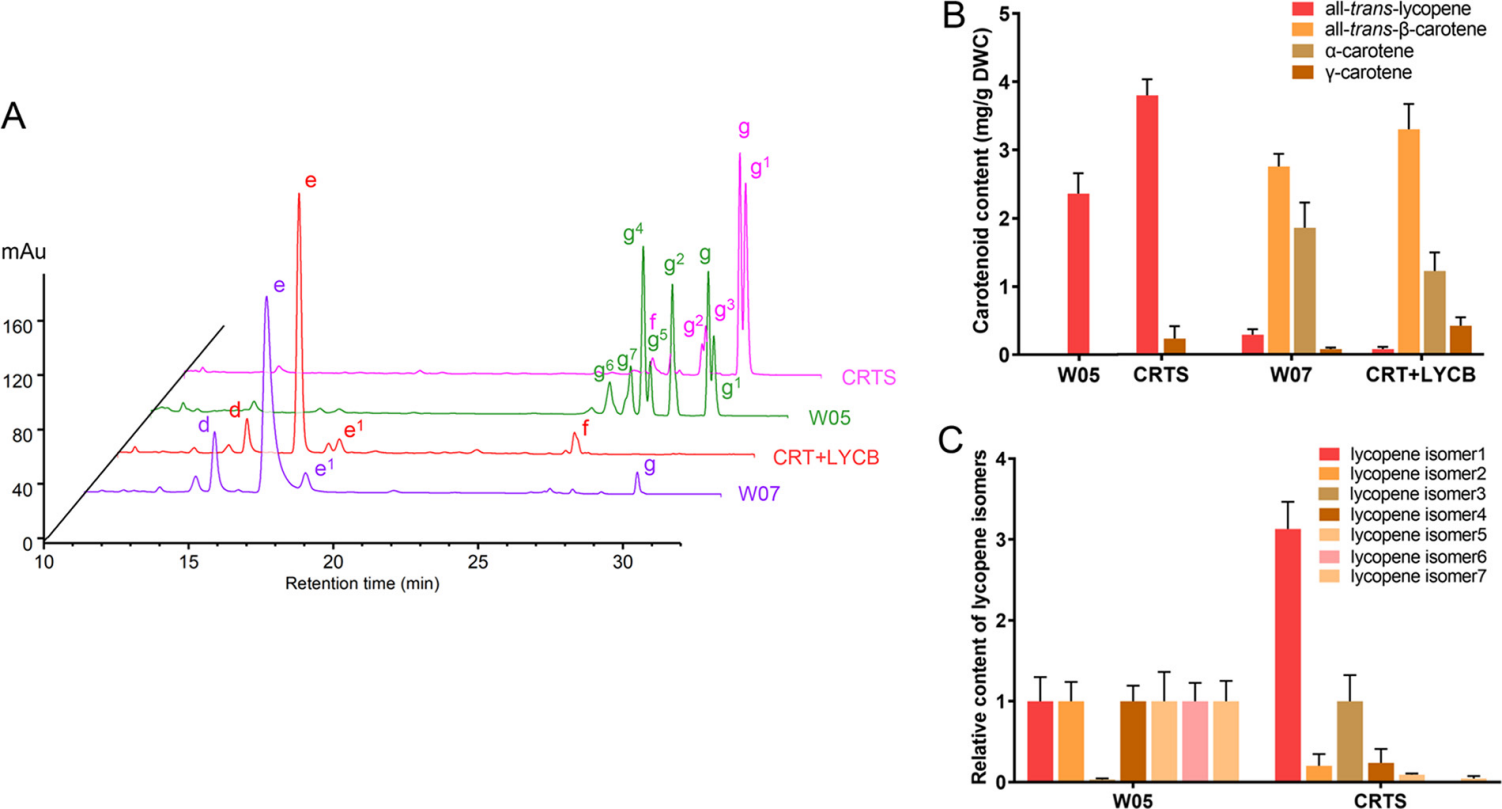
Comparison of carotenoid composition of algal and bacterial carotene synthesis pathway engineering Escherichia coli. (A) HPLC analysis of the carotenoids produced in E. coli cells harboring different pathways of plasmids; peaks: d, α-carotene; e, all-*trans*-β-carotene; e1, 9-*cis*-β-carotene; f, γ-carotene; g, all-*trans*-lycopene; g1, lycopene isomer1; g2, lycopene isomer2; g3, lycopene isomer3; g4, lycopene isomer4; g5, lycopene isomer5; g6, lycopene isomer6; g7, lycopene isomer7. (B) Carotenoid content in W05, CRTS, W07, and CRT+LYCB strains. (C) Relative content of lycopene isomers in W05 and CRTS strains.

The content of all-*trans*-β-carotene in the CRT+LYCB strain constructed in this study was 3.3 mg/g (cell dry weight) without optimization, while that in the engineered E. coli W07 strain constructed with the *Erwinia* gene was 2.75 mg/g (Fig. 7A and B). The content of all-*trans*-lycopene in CRTS strain was 3.8 mg/g, which was more than 1.6 times of that in *Erwinia* genetic engineered E. coli strain W05 (2.36 mg/g) (Fig. 7A and B). We note that many lycopene isomers were generated in the W05 strain, whereas our constructed CRTS strain mainly generated all-*trans*-lycopene (Fig. 7A and C). This indicates a higher specificity of the carotenoid synthesis pathway in algae.

In the step-by-step construction from DsGGPS to DsLYCB, the products were efficiently synthesized to the end products and their isomers of the cutoff step, while the contents of intermediate products and original substrates were few, such as lycopene, indicating that the catalytic efficiency of these enzymes was extremely high, which was probably one of the important reasons for the high accumulation of β-carotene in *D. salina.* This provides us with a more efficient pathway than the bacterial-derived carotenoid synthesis pathway (37, 38).

### Conclusion.

**FIG 8:**
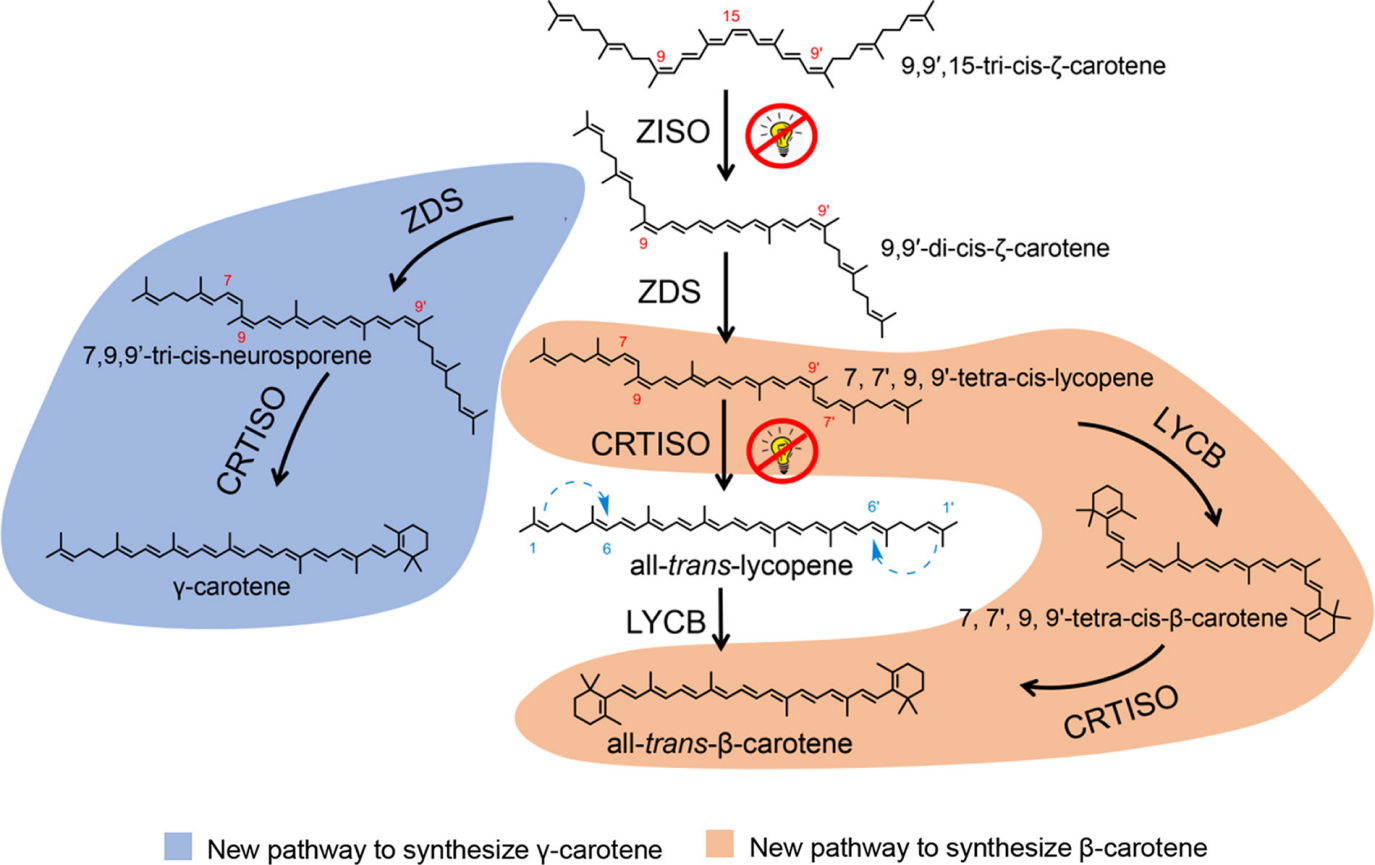
The β-carotene biosynthesis pathway in *Dunaliella*; blue area, new pathway to synthesize γ-carotene; orange area, new pathway to synthesize β-carotene.

Here, the *DsGGPS*, *DsPSY*, *DsPDS*, *DsZISO*, *DsZDS*, *DsCRTISO*, and *DsLYCB* genes from *D. salina* were successfully constructed, and highly expressed β-carotene engineering bacteria CRT+LYCB were obtained, confirming the β-carotene synthesis pathway of *D. salina*. Carotenoid synthesis of *D. salina* is highly similar to that of plants but has some differences. The functions of some enzymes have been redefined; in addition to the dehydrogenation function, DsZDS also has a cyclization function. DsLYCB also has catalytic activity for 7,9-*cis*-type lycopene, and is different from some plants, DsZISO and DsCRTISO cannot be replaced by photoisomerism. A new carotenoid 7,7′,9,9′-tetra-*cis*-β-carotene was discovered. These findings provided new ideas for the development of a high-yield carotenoid series of engineering bacteria (Fig. 8).

## MATERIALS AND METHODS

### Chemicals and reagents.

Carotenoid standards (β-carotene, all-*trans*-lycopene, α-carotene, γ-carotene, ε-carotene, δ-carotene, zeaxanthin, and lutein) were purchased from Sigma-Aldrich (USA) with purity ranging from 90% to 99%. 9,15,9′-Tri-*cis*-ζ-carotene and 9,9′-di-*cis*-ζ-carotene are carotenoid extracts from an engineered E. coli strain constructed from plasmids in the Addgene database pAC-ZETAipi (Addgene 53287) (39). The strain carrying the plasmids pACCRT-EIB and pACCAR16ΔcrtX, generously provided by Norihiko Misawa from Japan Ishikawa Prefectural University, can produce lycopene and β-carotene, respectively (40).

### RNA extraction and cDNA template preparation.

*D. salina* CCAP 19/18 cells were cultivated in a defined medium containing 1.5 M NaCl at 26°C and 8,000 lx provided under a 16-h light/8-h dark cycle with shaking at 96 rpm for about 14 days (exponential phase) (31). Total RNA was obtained from *D. salina* using the TRIzol reagent system (Invitrogen). First-strand cDNA was synthesized by PCR reverse transcription using the SuperScript III first-strand synthesis system (Thermo Fisher Scientific) with oligonucleotide (dT)_18_ primers and total RNA as a template. The 5′- and 3′-end sequences of the CDS were validated using SMARTer RACE cDNA amplification kit (Clontech).

### Sequence alignment and phylogenetic analyses.

Sequence alignment was performed using the MUSCLE algorithm of MEGA7 (41, 42). Based on the Jones-Taylor-Thornton (JTT) matrix-based model, evolutionary history was inferred using the maximum-likelihood method (43). Bootstrap values were obtained using 1,000 repeated calculations. All phylogenetic analyses were performed using MEGA7.

### Cloning of carotene synthesis genes from cDNA.

High-fidelity KOD DNA polymerase (TOYOBO) was used for PCR amplification using the cDNA of *D. salina* as a template. The amplified gene was cloned into the target vector using Gibson assembly (44). Restriction endonucleases were purchased from Thermo Fisher Scientific, and Gibson assembly reagents were purchased from New England BioLabs (NEB). Oligonucleotide primers were purchased from Sangon Biotech. Table S3 (available at https://www.biosynnatlab.com/wp-content/uploads/2022/12/Supplemental-Material.pdf) lists the primers used for cloning.

### Reconstitution of carotene biosynthesis in E. coli.

The plasmid construction schemes and maps of carotenoid engineering E. coli are shown in Fig. S5 at https://www.biosynnatlab.com/wp-content/uploads/2022/12/Supplemental-Material.pdf. The *crts* genes were inserted into pACYDuet-1, pETDuet-1, and pCDFDuet-1 (Novagen), respectively, using the Gibson assembly system. Primers for plasmid construction are shown in Table S3 at https://www.biosynnatlab.com/wp-content/uploads/2022/12/Supplemental-Material.pdf. The constructed vectors were respectively transformed into *E. coil* BL21(DE3) in the form of cotransformation to form a series of carotenoid engineered bacteria. The cotransformation scheme was shown in Table S4 at https://www.biosynnatlab.com/wp-content/uploads/2022/12/Supplemental-Material.pdf. The engineered E. coli single colonies were cultured in 50 mL of Luria-Bertani (LB) medium containing the corresponding antibiotics and shaken at 37°C at 200 rpm overnight. Overnight cultures were transferred to 250 mL of LB medium with corresponding antibiotics for expanded culture until an optical density at 600 nm (OD_600_) of 0.8 was reached. Expression was then induced with 1.0 mM isopropyl β-d-1-thiogalactopyranoside (IPTG) for 48 h in a light or dark environment at 28°C and 100 rpm.

### Carotenoids extraction from *D. salina* and E. coli.

Carotenoids are easily degraded and must be extracted under weak light conditions. Carotenoids in E. coli were extracted with cold acetone using a homogenizer for 20 min. After centrifugation, the carotenoid supernatant was collected. For the extraction of carotenoids from *D. salina*, cold acetone was added to the algal cells and dispersed on a homogenizer. After centrifugation, the supernatant was collected, and KOH solution was added until the final concentration reached 6%. After centrifugation at 10,000 rpm and 4°C, chlorophylls were extracted into the aqueous phase, and carotenoids were retained in the acetone phase. The extracted carotenoid solution of E. coli and *D. salina* was filtered with a 0.22-nm filter membrane and dried under a nitrogen evaporator. Then, the dried residue was redissolved in 200 μL of acetone.

### HPLC analysis of carotenoids.

Carotenoid analysis was performed using an Agilent 1260 HPLC system with a reversed-phase C30 YMC carotenoid column (5 μm, 250 × 4.6 mm) protected by a C30 guard column (5 μm, 10 × 4 mm) (YMC Co., Ltd., Japan) (20, 45). The solvent systems were A, consisting of methanol (MeOH)/water (97/3 [vol/vol]), 0.05 M ammonium acetate, and 0.1% butylated hydroxytoluene (BHT), and B, consisting of *tert*-butyl-methyl ether with 0.1% BHT. The following gradient elution was used: 0 min 90% A and 10% B, 0 to 10 min 60% A and 40% B, 10 to 20 min 50% A and 50% B, 20 to 25 min 10% A and 90% B, 25 to 29 min 10% and 90% B, 29 to 29.5 min 90% A and 10%, and 29.5 to 40 min 90% A and 10% B. The flow rate was 1 mL/min, and the injection volume was 10 μL. The detection of analytes was performed by UV absorbance at 450 nm and three-dimensional scanning from 200 to 700 nm.

## References

[1] Lamers PP, Janssen M, De Vos RC, Bino RJ, Wijffels RH. 2008. Exploring and exploiting carotenoid accumulation in *Dunaliella salina* for cell-factory applications. Trends Biotechnol 26:631–638. doi:10.1016/j.tibtech.2008.07.002. 18752860

[2] Chen H, Jiang JG. 2009. Osmotic responses of *Dunaliella* to the changes of salinity. J Cell Physiol 219:251–258. doi:10.1002/jcp.21715. 19202552

[3] Ye ZW, Jiang JG, Wu GH. 2008. Biosynthesis and regulation of carotenoids in *Dunaliella*: progresses and prospects. Biotechnol Adv 26:352–360. doi:10.1016/j.biotechadv.2008.03.004. 18486405

[4] Sun T, Yuan H, Cao H, Yazdani M, Tadmor Y, Li L. 2018. Carotenoid metabolism in plants: the role of plastids. Mol Plant 11:58–74. doi:10.1016/j.molp.2017.09.010. 28958604

[5] Rodriguez-Concepcion M, Avalos J, Bonet ML, Boronat A, Gomez-Gomez L, Hornero-Mendez D, Limon MC, Melendez-Martinez AJ, Olmedilla-Alonso B, Palou A, Ribot J, Rodrigo MJ, Zacarias L, Zhu C. 2018. A global perspective on carotenoids: metabolism, biotechnology, and benefits for nutrition and health. Prog Lipid Res 70:62–93. doi:10.1016/j.plipres.2018.04.004. 29679619

[6] Steinwand MA, Ronald PC. 2020. Crop biotechnology and the future of food. Nat Food 1:273–283. doi:10.1038/s43016-020-0072-3.

[7] Huang JJ, Lin S, Xu W, Cheung PCK. 2017. Occurrence and biosynthesis of carotenoids in phytoplankton. Biotechnol Adv 35:597–618. doi:10.1016/j.biotechadv.2017.05.001. 28511892

[8] Wang P, Wang Y, Wang W, Chen T, Tian S, Qin G. 2020. Ubiquitination of phytoene synthase 1 precursor modulates carotenoid biosynthesis in tomato. Commun Biol 3:730. doi:10.1038/s42003-020-01474-3. 33273697 PMC7713427

[9] Gong M, Bassi A. 2016. Carotenoids from microalgae: a review of recent developments. Biotechnol Adv 34:1396–1412. doi:10.1016/j.biotechadv.2016.10.005. 27816618

[10] Liang MH, Lu Y, Chen HH, Jiang JG. 2017. The salt-regulated element in the promoter of lycopene β-cyclase gene confers a salt regulatory pattern in carotenogenesis of *Dunaliella bardawil*. Environ Microbiol 19:982–989. doi:10.1111/1462-2920.13539. 27657551

[11] Liang MH, Liang YJ, Jin HH, Jiang JG. 2015. Characterization and functional identification of a gene encoding geranylgeranyl diphosphate synthase from *Dunaliella bardawil*. J Agric Food Chem 63:7805–7812. doi:10.1021/acs.jafc.5b02732. 26289929

[12] Ye X, Al-Babili S, Kloti A, Zhang J, Lucca P, Beyer P, Potrykus I. 2000. Engineering the provitamin A (beta-carotene) biosynthetic pathway into (carotenoid-free) rice endosperm. Science 287:303–305. doi:10.1126/science.287.5451.303. 10634784

[13] Paine JA, Shipton CA, Chaggar S, Howells RM, Kennedy MJ, Vernon G, Wright SY, Hinchliffe E, Adams JL, Silverstone AL, Drake R. 2005. Improving the nutritional value of Golden Rice through increased pro-vitamin A content. Nat Biotechnol 23:482–487. doi:10.1038/nbt1082. 15793573

[14] Polle JEW, Barry K, Cushman J, Schmutz J, Tran D, Hathwaik LT, Yim WC, Jenkins J, McKie-Krisberg Z, Prochnik S, Lindquist E, Dockter RB, Adam C, Molina H, Bunkenborg J, Jin E, Buchheim M, Magnuson J. 2017. Draft nuclear genome sequence of the halophilic and beta-carotene-accumulating green alga *Dunaliella salina* strain CCAP19/18. Genome Announc 5:e01105-17. doi:10.1128/genomeA.01105-17. 29074648 PMC5658486

[15] Goodstein DM, Shu S, Howson R, Neupane R, Hayes RD, Fazo J, Mitros T, Dirks W, Hellsten U, Putnam N, Rokhsar DS. 2012. Phytozome: a comparative platform for green plant genomics. Nucleic Acids Res 40:D1178–D1186. doi:10.1093/nar/gkr944. 22110026 PMC3245001

[16] Liang M-H, Hao Y-F, Li Y-M, Liang Y-J, Jiang J-G. 2016. Inhibiting lycopene cyclases to accumulate lycopene in high β-carotene-accumulating *Dunaliella bardawil*. Food Bioproc Tech 9:1002–1009. doi:10.1007/s11947-016-1681-6.

[17] Ramos A, Coesel S, Marques A, Rodrigues M, Baumgartner A, Noronha J, Rauter A, Brenig B, Varela J. 2008. Isolation and characterization of a stress-inducible *Dunaliella salina* Lcy-β gene encoding a functional lycopene β-cyclase. Appl Microbiol Biotechnol 79:819–828. doi:10.1007/s00253-008-1492-4. 18461318

[18] Cunningham FX, Jr, Pogson B, Sun Z, McDonald KA, DellaPenna D, Gantt E. 1996. Functional analysis of the beta and epsilon lycopene cyclase enzymes of *Arabidopsis* reveals a mechanism for control of cyclic carotenoid formation. Plant Cell 8:1613–1626. doi:10.1105/tpc.8.9.1613. 8837512 PMC161302

[19] Chen X, Han H, Jiang P, Nie L, Bao H, Fan P, Lv S, Feng J, Li Y. 2011. Transformation of β-lycopene cyclase genes from *Salicornia europaea* and *Arabidopsis* conferred salt tolerance in *Arabidopsis* and tobacco. Plant Cell Physiol 52:909–921. doi:10.1093/pcp/pcr043. 21471119

[20] Liang MH, Liang ZC, Chen HH, Jiang JG. 2019. The bifunctional identification of both lycopene β- and ε-cyclases from the lutein-rich *Dunaliella bardawil*. Enzyme Microb Technol 131:109426. doi:10.1016/j.enzmictec.2019.109426. 31615667

[21] Bai L, Kim EH, DellaPenna D, Brutnell TP. 2009. Novel lycopene epsilon cyclase activities in maize revealed through perturbation of carotenoid biosynthesis. Plant J 59:588–599. doi:10.1111/j.1365-313X.2009.03899.x. 19392686

[22] Stickforth P, Steiger S, Hess WR, Sandmann G. 2003. A novel type of lycopene ε-cyclase in the marine cyanobacterium *Prochlorococcus marinus* MED4. Arch Microbiol 179:409–415. doi:10.1007/s00203-003-0545-4. 12712234

[23] Mialoundama AS, Heintz D, Jadid N, Nkeng P, Rahier A, Deli J, Camara B, Bouvier F. 2010. Characterization of plant carotenoid cyclases as members of the flavoprotein family functioning with no net redox change. Plant Physiol 153:970–979. doi:10.1104/pp.110.155440. 20460582 PMC2899934

[24] Yu Q, Ghisla S, Hirschberg J, Mann V, Beyer P. 2011. Plant carotene *cis*-*trans* isomerase CRTISO a new member of the FAD(RED)-dependent flavoproteins catalyzing non-redox reactions. J Biol Chem 286:8666–8676. doi:10.1074/jbc.M110.208017. 21209101 PMC3048748

[25] Chen Y, Li F, Wurtzel ET. 2010. Isolation and characterization of the Z-ISO gene encoding a missing component of carotenoid biosynthesis in plants. Plant Physiol 153:66–79. doi:10.1104/pp.110.153916. 20335404 PMC2862425

[26] Sugiyama K, Takahashi K, Nakazawa K, Yamada M, Kato S, Shinomura T, Nagashima Y, Suzuki H, Ara T, Harada J, Takaichi S. 2020. Oxygenic phototrophs need zeta-carotene isomerase (Z-ISO) for carotene synthesis: functional analysis in *Arthrospira* and *Euglena*. Plant Cell Physiol 61:276–282. doi:10.1093/pcp/pcz192. 31593237

[27] Fantini E, Falcone G, Frusciante S, Giliberto L, Giuliano G. 2013. Dissection of tomato lycopene biosynthesis through virus-induced gene silencing. Plant Physiol 163:986–998. doi:10.1104/pp.113.224733. 24014574 PMC3793073

[28] Dong H, Deng Y, Mu J, Lu Q, Wang Y, Xu Y, Chu C, Chong K, Lu C, Zuo J. 2007. The *Arabidopsis spontaneous cell death1* gene, encoding a zeta-carotene desaturase essential for carotenoid biosynthesis, is involved in chloroplast development, photoprotection and retrograde signalling. Cell Res 17:458–470. doi:10.1038/cr.2007.37. 17468780

[29] Avendano-Vazquez AO, Cordoba E, Llamas E, San Roman C, Nisar N, De la Torre S, Ramos-Vega M, Gutierrez-Nava MD, Cazzonelli CI, Pogson BJ, Leon P. 2014. An uncharacterized apocarotenoid-derived signal generated in zeta-carotene desaturase mutants regulates leaf development and the expression of chloroplast and nuclear genes in *Arabidopsis*. Plant Cell 26:2524–2537. doi:10.1105/tpc.114.123349. 24907342 PMC4114949

[30] Bartley GE, Scolnik PA, Beyer P. 1999. Two *Arabidopsis thaliana* carotene desaturases, phytoene desaturase and zeta-carotene desaturase, expressed in *Escherichia coli*, catalyze a poly-*cis* pathway to yield pro-lycopene. Eur J Biochem 259:396–403. doi:10.1046/j.1432-1327.1999.00051.x. 9914519

[31] Lao YM, Xiao L, Luo LX, Jiang JG. 2014. Hypoosmotic expression of *Dunaliella bardawil* ζ-carotene desaturase is attributed to a hypoosmolarity-responsive element different from other key carotenogenic genes. Plant Physiol 165:359–372. doi:10.1104/pp.114.235390. 24632600 PMC4012594

[32] Brown HC, Brewster J, Shechter H. 1954. An interpretation of the chemical behavior of five- and six-membered ring compounds. J Am Chem Soc 76:467–474. doi:10.1021/ja01631a041.

[33] Fleetham TB, Huang L, Klimes K, Brooks J, Li J. 2016. Tetradentate Pt (II) complexes with 6-membered chelate rings: a new route for stable and efficient blue organic light emitting diodes. Chem Mater 28:3276–3282. doi:10.1021/acs.chemmater.5b04957.

[34] Sandmann G. 2002. Molecular evolution of carotenoid biosynthesis from bacteria to plants. Physiol Plant 116:431–440. doi:10.1034/j.1399-3054.2002.1160401.x.

[35] Furubayashi M, Saito K, Umeno D. 2014. Evolutionary analysis of the functional plasticity of *Staphylococcus aureus* C30 carotenoid synthase. J Biosci Bioeng 117:431–436. doi:10.1016/j.jbiosc.2013.10.003. 24216462

[36] Breitenbach J, Bruns M, Sandmann G. 2013. Contribution of two ζ-carotene desaturases to the poly-*cis* desaturation pathway in the cyanobacterium *Nostoc* PCC 7120. Arch Microbiol 195:491–498. doi:10.1007/s00203-013-0899-1. 23695436

[37] Zhao J, Li Q, Sun T, Zhu X, Xu H, Tang J, Zhang X, Ma Y. 2013. Engineering central metabolic modules of *Escherichia coli* for improving β-carotene production. Metab Eng 17:42–50. doi:10.1016/j.ymben.2013.02.002. 23500001

[38] Misawa N, Nakagawa M, Kobayashi K, Yamano S, Izawa Y, Nakamura K, Harashima K. 1990. Elucidation of the *Erwinia uredovora* carotenoid biosynthetic pathway by functional analysis of gene products expressed in *Escherichia coli*. J Bacteriol 172:6704–6712. doi:10.1128/jb.172.12.6704-6712.1990. 2254247 PMC210783

[39] Cunningham FX, Jr, Gantt E. 2007. A portfolio of plasmids for identification and analysis of carotenoid pathway enzymes: *Adonis aestivalis* as a case study. Photosynth Res 92:245–259. doi:10.1007/s11120-007-9210-0. 17634749

[40] Misawa N, Satomi Y, Kondo K, Yokoyama A, Kajiwara S, Saito T, Ohtani T, Miki W. 1995. Structure and functional analysis of a marine bacterial carotenoid biosynthesis gene cluster and astaxanthin biosynthetic pathway proposed at the gene level. J Bacteriol 177:6575–6584. doi:10.1128/jb.177.22.6575-6584.1995. 7592436 PMC177511

[41] Edgar RC. 2004. MUSCLE: multiple sequence alignment with high accuracy and high throughput. Nucleic Acids Res 32:1792–1797. doi:10.1093/nar/gkh340. 15034147 PMC390337

[42] Kumar S, Stecher G, Tamura K. 2016. MEGA7: molecular evolutionary genetics analysis version 7.0 for bigger datasets. Mol Biol Evol 33:1870–1874. doi:10.1093/molbev/msw054. 27004904 PMC8210823

[43] Jones DT, Taylor WR, Thornton JM. 1992. The rapid generation of mutation data matrices from protein sequences. Comput Appl Biosci 8:275–282. doi:10.1093/bioinformatics/8.3.275. 1633570

[44] Gibson DG, Young L, Chuang RY, Venter JC, Hutchison CA, III, Smith HO. 2009. Enzymatic assembly of DNA molecules up to several hundred kilobases. Nat Methods 6:343–345. doi:10.1038/nmeth.1318. 19363495

[45] Liang MH, Xie H, Chen HH, Liang ZC, Jiang JG. 2020. Functional identification of two types of carotene hydroxylases from the green alga *Dunaliella bardawil* rich in lutein. ACS Synth Biol 9:1246–1253. doi:10.1021/acssynbio.0c00070. 32408742

[46] Guo W, Liu Y, Yan X, Liu M, Tang H, Liu Z, Zhang L. 2015. Cloning and characterization of a phytoene dehydrogenase gene from marine yeast *Rhodosporidium diobovatum*. Antonie Van Leeuwenhoek 107:1017–1027. doi:10.1007/s10482-015-0394-6. 25627014

[47] Harada J, Nagashima KV, Takaichi S, Misawa N, Matsuura K, Shimada KJP. 2001. Phytoene desaturase, CrtI, of the purple photosynthetic bacterium, *Rubrivivax gelatinosus*, produces both neurosporene and lycopene. Plant Cell Physiol 42:1112–1118. doi:10.1093/pcp/pce140. 11673627

[48] Hausmann A, Sandmann G. 2000. A single five-step desaturase is involved in the carotenoid biosynthesis pathway to β-carotene and torulene in *Neurospora crassa*. Fungal Genet Biol 30:147–153. doi:10.1006/fgbi.2000.1212. 11017770

[49] Iniesta AA, Cervantes M, Murillo FJ. 2007. Cooperation of two carotene desaturases in the production of lycopene in *Myxococcus xanthus*. FEBS J 274:4306–4314. doi:10.1111/j.1742-4658.2007.05960.x. 17662111

[50] Frigaard N-U, Maresca JA, Yunker CE, Jones AD, Bryant DA. 2004. Genetic manipulation of carotenoid biosynthesis in the green sulfur bacterium *Chlorobium tepidum*. J Bacteriol 186:5210–5220. doi:10.1128/JB.186.16.5210-5220.2004. 15292122 PMC490927

